# Application of open-door laminoplasty with ARCH plate fixation in cervical intraspinal tumors

**DOI:** 10.1186/s12893-021-01140-3

**Published:** 2021-03-19

**Authors:** Zhi-Chao Wang, Shu-Zhong Li, Xin-Fei Qu, Chu-Qiang Yin, Yuan-Liang Sun, Yue-Lei Wang, Jie Wang, Chen-Jing Liu, Zhen-Lu Cao, Ting Wang

**Affiliations:** 1 Department of Spine Surgery, Affiliated Hospital of Qingdao University, Jiangsu Road, Shinan District, Qingdao, 266000 China; 2 Department of Operating Room, Affiliated Hospital of Qingdao University, Qingdao, 266000 China

**Keywords:** ARCH plate, Cervical, Open-door laminoplasty, Intradural tumor

## Abstract

**Background:**

The open-door laminoplasty is an effective procedure for the treatment of cervical spondylotic myelopathy. However, little information is available about the surgical results of open-door laminoplasty in the treatment of intraspinal tumors. In the present study, we aimed to investigate the clinical effect of open-door laminoplasty with ARCH plate fixation in the treatment of cervical intraspinal tumors.

**Methods:**

This was a retrospective study. From January 2013 to May 2018, 38 patients (13 males and 25 females, the average age of 44 ± 17 years) with cervical intraspinal tumors underwent open-door laminoplasty with ARCH plate fixation in our hospital. The operation time, blood loss, pre- and postoperative visual analog scale (VAS), and Japanese Orthopedic Association (JOA) scores were determined. To determine the radiographic outcomes, cervical X-ray film and magnetic resonance imaging (MRI) were performed before and after the operation, and cervical X-ray sagittal film was used to measure Cobb angle. The clinical data before and after the operation were compared by t-test.

**Results:**

A total of 38 patients underwent a successful operation and demonstrated primary healing. The average operation time was 113 ± 12 min. The average blood loss was 120 ± 19 mL. All patients were followed up for 26.1 ± 2.8 months, and the final follow-up time was more than 24 months. VAS scores were much better at 24 months after operation compared with those before the operation, which were decreased from 6.1 ± 1.1 to 1.4 ± 0.7 (t = 32.63, P < 0.01). The JOA score was improved from 9.9 ± 1.5 to 15.5 ± 0.6 (t = − 18.36, P < 0.01), and the mean JOA recovery rate was 79% ± 11% at 24 months after the operation. There was no significant difference in Cobb angle between pre-operation and 24 months after the operation, which was 9.8 ± 2.6 and 10.3 ± 3.1 respectively (t = − 0.61, P > 0.05). Neither spinal malalignment on the coronal plane nor displacement of the laminoplasty flap was observed on postoperative cervical X-ray and MRI examinations at the final follow-up.

**Conclusions:**

Open-door laminoplasty with ARCH plate fixation was a safe and effective surgical approach for the treatment of cervical intraspinal tumors.

## Background

Due to the compression of intraspinal tumors, spinal cord and nerve roots may have severe dysfunction. The most common procedure for intraspinal tumors is the classical total laminectomy. The classical total laminectomy provides patients with sufficient operative exposure for the safe extirpation of spinal tumor. However, the invasion of hematoma and scar tissue into the spinal canal is the potential problems. The lamina, facet joint, supraspinous ligament, interspinous ligament, and ligamentum flavum are the important components of the posterior column of the spine. The biomechanical stability of the spine is most affected by the removal of the posterior column structure of the spine. This situation usually leads to spinal structure instability and kyphosis, especially for patients with cervical intraspinal tumors [1–3]. Zong et al. [4] have reported that the total laminectomy and screw-rod internal fixation significantly reduced the incidence of spinal structural instability and kyphosis to some extent, comparing with the classical total laminectomy, in short-term follow-up. However, due to the loss of posterior bone structure, the scar tissue is still the risk of spinal cord compression after operation [1, 5, 6].

At present, the importance of rebuilding the stability of the spine is recognized by more and more surgeons. Laminoplasty has the obvious advantage of reducing the postoperative risks of classical total laminectomy by rebuilding the integrity of the posterior elements. Following the first report of lamina replantation by Raimondi in 1976 [7], a variety of laminoplasty, with a good satisfactory surgical results, has been used to treat patients with intraspinal tumors [5, 6, 8–10]. In addition, because the laminoplasty retains the relatively normal posterior structure, it can help avoid the epidural scar adhesion and contraction of the dura mater. Laminoplasty also makes revision surgery easier and safer than laminectomy when intradural tumors recur [6].

Among them, although Iplikcioglu et al. [10] have reported that 13 patients with intraspinal tumors are treated with open-door laminoplasty, the patients are mainly associated with thoracic or lumbar intraspinal tumors. In the present study, we aimed to investigate the clinical effect of open-door laminoplasty with ARCH plate fixation in the treatment of cervical intraspinal tumors.

## Methods

### Patients

**Fig. 1:**
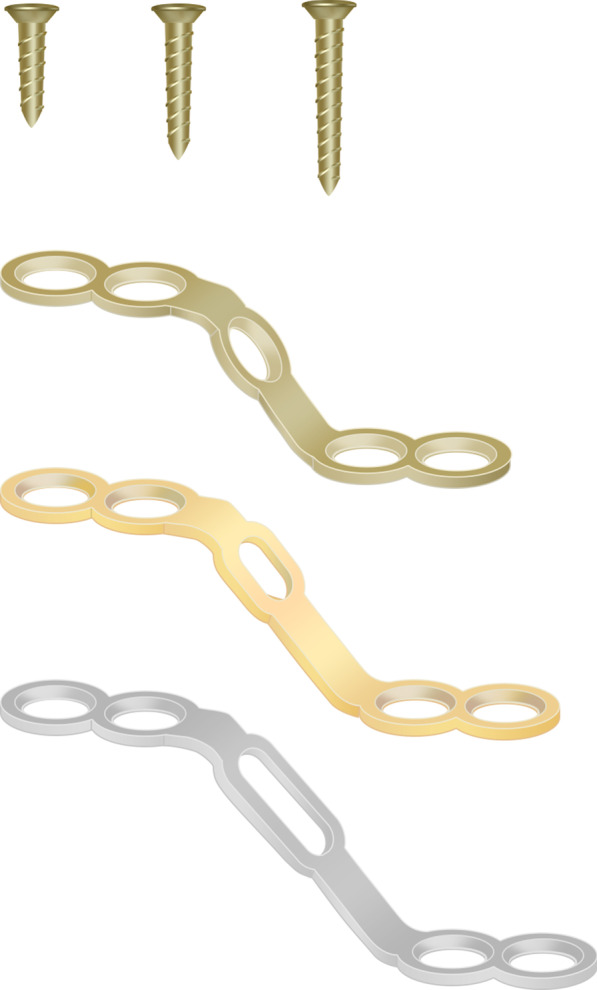
Arch plates and titanium screws used during operation. Hand-drawn schematic diagram of ARCH plate and titanium screws

This study involved a retrospective design. The participants consisted of 38 patients who underwent the open-door laminoplasty with ARCH plate (Johnson & Johnson Inc. America) (Fig. 1) fixation to treat cervical intraspinal tumors between January 2013 to May 2018. All patients were diagnosed by conducting physical examinations using cervical X-ray, computed tomography (CT), and magnetic resonance imaging (MRI). After the lesions were found by routine scan, enhanced scanning was performed to clarify the scope and size of the tumor as well as its relationship with the spinal cord, cauda equina, and nerve root. All patients who met the following criteria were included in the study: (1) all patients with intraspinal tumors underwent an operation for the first time; (2) no spinal structure destruction or spine instability; (3) clinical and pathological data were complete; (4) all patients were followed up for at least 2 years. Exclusion criteria were as follows: (1) facet joint was destroyed, (2) patient suffered from tumor recurrence, and (3) lesions led to spinal destruction and instability. This study was conducted following the Declaration of Helsinki, and approved by the Ethics Committee of the Affiliated Hospital of Qingdao University (Qingdao, China). Written informed consent was obtained from all participants.

### Surgical technique

**Fig. 2:**
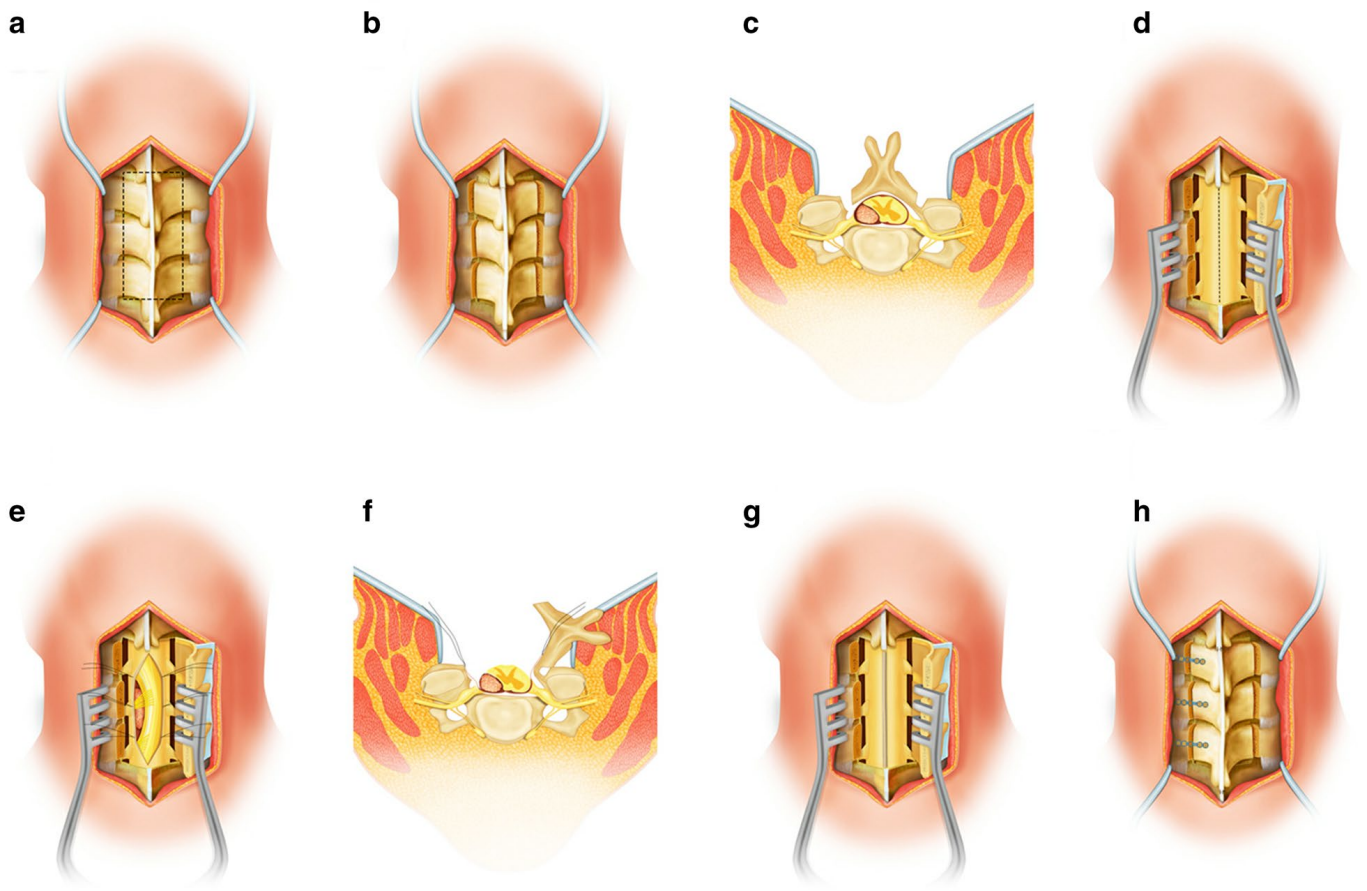
Hand-drawn schematic diagram of surgical procedure **a** The paraspinal muscles were dissected and separated under the periosteum to expose the spinous process and lamina, while the supraspinous and interspinous ligaments remained intact. **b**, **c** Minimal laminotomy was performed on the open side, and a green stick fracture was formed on the opposite side. **d** A stretcher was used to expose the visual field of the operation. **e**, **f** A longitudinal incision was made at the center of the posterior dura mater under a microscope and pulled open with silk thread. Complete separation and resection of the tumor were performed to decompress the spinal cord. **g** Waterproof suture was performed on the dura mater after tumor resection. **h** The lamina was reduced and fixed with titanium screw and ARCH plate. Moreover, the suture line of tendon was used to fix the supraspinous ligament of the head and tail of the lamina

The patients were administered with general anesthesia through tracheal intubation, and the operation was performed in the prone position. A median incision was made according to the location of the tumor, which was determined by MRI before the operation, and the paraspinal muscles were dissected and separated under the periosteum to carefully preserve the facet joint capsule. The spinous process and vertebral lamina were exposed, while the supraspinous and interspinous ligaments remained intact (Fig. 2a). A groove was first made with a grinding drill (2–3 mm), and then a minimum laminotomy was performed using fine rongeurs (1 mm) inside the facet joints of the open side. The decision of laminotomy on one side was made based on the location of the lesions in the spinal canal. For laterally placed lesions, the laminotomy was performed on the same side. For lesions located in the center of the spinal canal, laminotomy was performed on the left side. On the opposite side, a grinding drill was used to make grooves on the medial side of the facet joint and the lateral cortical bone of the lamina (Fig. 2b, c), and the supraspinous ligament and interspinous ligament of the head and tail segments of the lamina were cut off. The lamina was lifted, the spinous process was pushed back to create a contralateral green stick laminar fracture, and the laminae were then placed as a block over the paravertebral muscles. Moreover, a spreader was used to expose the visual field of the operation (Fig. 2d). Subsequently, a longitudinal incision was made at the center of the posterior dura mater under a microscope and pulled to open with silk thread. Complete separation and resection of the tumor were carried out to decompress the spinal cord (Fig. 2e, f). A waterproof suture was used on the dura mater after tumor resection (Fig. 2g). Next, the lamina was reduced. After holes were made in the spinous process and lateral mass, they were fixed with titanium screws and ARCH plates. The suture line of the tendon was used to fix the supraspinous ligament of the head and tail of the lamina to further maintain the replantation of the lamina in situ (Fig. 2h). The tube placement was drained, and then the incision was washed and closed at each layer.

### Postoperative management

Antibiotics were used prophylactically for 48 h after the operation, and the deterioration of neurological symptoms and drainage were observed. If there was no cerebrospinal fluid (CSF) leakage, the patient could pull out the drainage tube 24 h after the operation. If there was CSF leakage, CSF drainage was controlled within 150–200 mL every day until the drainage was clear. The drainage tube was pulled out, and the wound was pressurized and bandaged [11]. The patient was allowed to get out of bed under the protection of a neck brace. The neck brace was removed 1 month after the operation.

### Outcome evaluation

#### Clinical data

All patients were followed up for at least 2 years. The operation time, blood loss, pathological type of tumor, and any postoperative complications were recorded. Neck pain was evaluated using a 10-point visual analog scale (VAS) preoperatively as well as 3 months, 12 months and 24 months post-operatively. The neurological status was evaluated using the Japanese Orthopedic Association (JOA) scores preoperatively as well as 3 months, 12 months and 24 months post-operatively. The JOA recovery rate at 24 months postoperatively, which represents the degree of normalization after surgery, was calculated using the Hirabayashi formula: (Postoperative score − Preoperative score) × 100/(17 − Preoperative score).

#### Radiographic data

To determine the level of internal fixation and spinal stability, cervical X-ray film examination was performed under the upright conditions at 1 day before the operation, as well as 1, 3, 6, 12 months, and annually post-operation. The cervical X-ray sagittal film in the upright state was used to measure the Cobb angle, which was defined by measuring the angle between the horizontal line at the lower edge of the C2 and C7 vertebrae. When the change between preoperative and postoperative angles was more than 10°, the operative segment was considered to be unstable. At 12 months after the surgery, MRI was performed to detect tumor recurrence and scar oppression in the spinal canal, as well as the repair of the ligaments. All radiologic measurements were reviewed by two experienced spinal surgeons.

### Statistical analysis

Data were presented as means ± standard deviation (SD). A paired t-test was used to statistically analyze the difference between the preoperative and postoperative scores. A P value of < 0.05 or < 0.01 was considered statistically significant.

## Results

The participants included 13 males and 25 females, and their ages ranged from 14 to 75 years (mean = 44 ± 17 years). The tumors involved in two, three, and four vertebral segments were found in 18 patients, 14 patients, and six patients, respectively. A total of 102 segments underwent laminoplasty. All patients were followed up for 26.1 ± 2.8 months, and the final follow-up time was more than 24 months.

### Clinical evaluation

All patients underwent a successful operation and demonstrated primary healing. The average operation time was 113 ± 12 min. The average blood loss was 120 ± 19 mL. Histological diagnoses were schwannoma in 25 patients, and ependymoma in seven patients, while meningioma or ganglioneuroma was respectively found in three patients. The pain and numbness were significantly relieved or disappeared following the operation. The VAS scores were much better 24 months after the operation compared with those before the operation, and they were decreased from 6.1 ± 1.1 to 1.4 ± 0.7 (t = 32.63, P < 0.01). At 24 months after the operation, the JOA score was improved from 9.9 ± 1.5 to 15.5 ± 0.6, attaining significant improvement (t = − 18.36, P < 0.01) (Table 1). The JOA recovery rate at 24 months after the operation was 79% ± 11%.

**Table 1:** Preoperative and postoperative data of patients

Items	Pre-operation	3 Month after operation	12 Months after operation	24 Months after operation	Pre-operation vs 24 months after operation	
	Mean (SD)	Mean (SD)	Mean (SD)	Mean (SD)	t	P
VAS	6.1 ± 1.1	2.1 ± 0.9	1.5 ± 0.8	1.4 ± 0.7^*^	32.63	< 0.0001
JOA scores	9.9 ± 1.5	13 ± 1.1	15.1 ± 0.8	15.5 ± 0.6^*^	− 18.36	< 0.0001
Cobb angle (°)	9.8 ± 2.6	9.7 ± 2.1	10.1 ± 2.7	10.3 ± 3.1	− 0.61	0.581

*VAS* visual analog scales, *JOA* Japanese Orthopedic Association scores, *Cobb Angle* the angle between the horizontal line at the lower edge of the C2 and C7 vertebrae

*P < 0.01 vs pre-operative score

### Radiologic evaluation

Neither spinal malalignment on the coronal plane nor displacement of the bone flap was observed on postoperative cervical X-ray film examinations. There was no significant difference in Cobb angle before and 24 months after operation (t = − 0.61, P > 0.05) (Table 1). At 12 months after the operation, MRI showed that the supraspinatus ligament was repaired and no tumor recurrence and epidural scar.

### Illustrative case

**Fig. 3:**
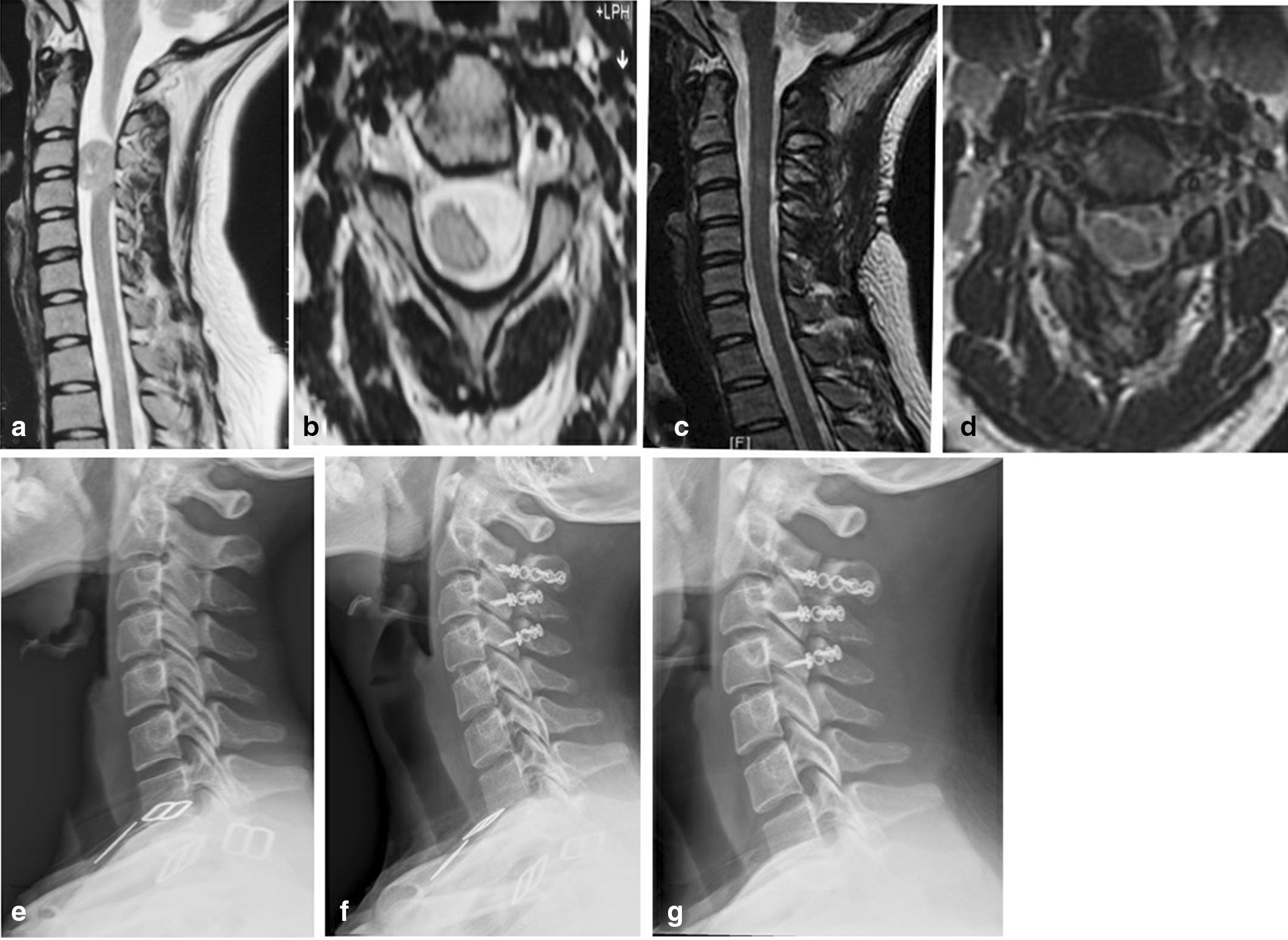
Case 3. The schwannoma at the C3-4 level. **a**, b Prior to surgery, MRI identified that the C3-4 spinal space was occupied by the tumor. **c**, **d** At 12 months after the surgery, no intraspinal scar adhesions or restenosis were identified by MRI. **e** Prior to surgery, cervical X-ray examination was performed under the upright conditions. **f**, **g** At 12 months and 24 months after the surgery, X-ray examination indicated no fixation transposition or fracture, cervical instability or kyphosis

**Fig. 4:**
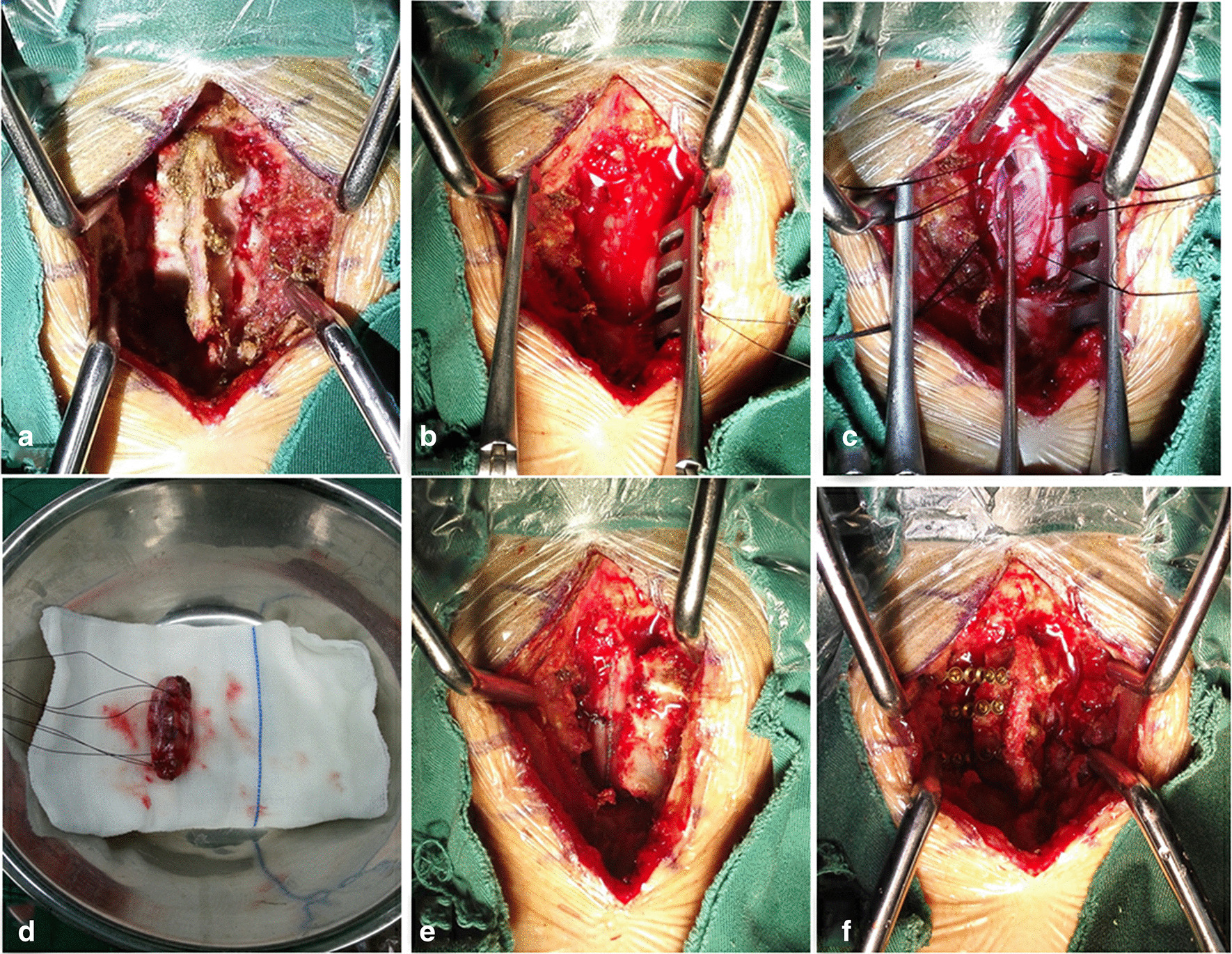
Case 3. **a** The paraspinal muscles were dissected and separated under the periosteum to expose the spinous process and lamina, while the supraspinous and interspinous ligaments remained intact. **b** The visual field of the operation was exposed. **c** The tumor was completely separated and removed under the microscope. **d** A completely resected tumor. **e** Waterproof suture was performed on the dura mater after tumor resection. **f** The lamina was reduced and fixed with titanium screw and ARCH plate. Moreover, the suture line of tendon was used to fix the supraspinous ligament of the head and tail of the lamina

Case 3. The patient had neck and shoulder pain as well as left upper limb numbness for 3 years. Admission for physical examination: left upper limb skin hyperalgesia and left upper limb muscle strength III-IV grade. Hoffmann sign ( +) and Babinski sign ( +). Cervical MRI showed that the intraspinal space was occupied by a lesion at C3–4 levels (Fig. 3a, b). The preoperative VAS score was 6, the JOA score was 8, and the Cobb angle was 9° (Fig. 3e). Open-door laminoplasty in combination with ARCH plate fixation was given for the treatment of intraspinal lesion (Fig. 4). After the operation, the postoperative pathological diagnosis was schwannoma. At 1 month after the operation, the neck and shoulder pain disappeared, the numbness and weakness of the left upper limb were relieved, and there were no complications, such as CSF leakage. At 3 months after the operation, the VAS score was 3, and the JOA score was 13. At 12 months after the operation, the VAS score was 1, the JOA score was 15. At 24 months after the operation, the VAS score was 1, the JOA score was 15 and the JOA recovery rate was 78%. At 12 months after the operation, the cervical MRI showed that the ligaments were repaired and healed (Fig. 3c, d). There were no intraspinal scar adhesions or restenosis. At 12 months after the operation, the cervical X-ray plain showed that the Cobb angle was 12° (Fig. 3f). At 24 months after the operation, the cervical X-ray plain showed that the Cobb angle was 14° (Fig. 3g).

## Discussion

Intradural extramedullary spinal tumors account for two-thirds of all intraspinal neoplasms [12]. Among them, three-fourths of intradural extramedullary spinal tumors are benign tumors with progressive aggravation [13]. Due to the risk of oppressing the spinal cord and nerve root, early tumor resection and spinal cord decompression can improve neurological function, reduce postoperative complications and decrease the risk of surgical operation [14]. According to the position relationship between spinal cord and tumor, the surgical approach is generally divided into the posterior approach, anterior approach, and combination of anterior and posterior approach. However, the anterior approach is difficult to expose and easy to damage blood vessels and nerves. Besides, the incidence of intraspinal tumors in the dorsal spinal cord is much higher than that in the ventral spinal cord. Therefore, the posterior approach has become a routine surgical approach [8].

The safe excision of spinal tumors depends on sufficient visualization of the tumor and surrounding structures [15]. Historically, classical total laminectomy has been used as the most common approach. However, according to the Denis's "three-column theory" of the spine, lamina, facet joint, spinous process, ligamentum flavum, interspinous ligament and supraspinous ligament are the important components of the posterior column of the spine, taking 24 to 30% pressure and 21 to 54% rotational stress [16, 17]. But the classical total laminectomy completely removes the posterior column structure of the spine. This surgical method may provide surgeon with the wider area of the spine during surgery, helping the complete resection of intraspinal tumor. But a series of complications, associating with weak bone protection, include postoperative spinal instability, kyphosis and symptomatic epidural scar formation. Among them, the most common complication is kyphosis [1–3, 18, 19], especially for patients with cervical intraspinal tumors. At the same time, Jiang et al. [20] indicated that supraspinous and interspinous ligaments are rich in nerve fibers and histologically proved to be afferent nerve fibers. For the existence of ligaments, the contraction of neck and back muscles can be regulated by the ligament-nerve-muscle nerve reflex system leading to the fine movement and posture of the spine [21].

Therefore, the basic principle of intraspinal tumor operation not only is the complete removal of intraspinal tumors but also the protection of spinal anatomical structure and function [22]. A variety of modified laminectomy and laminoplasty have been introduced as an alternative option of total laminectomy. Several reported methods include the hemi-laminectomy and hemi-laminoplasty [8, 23–25], process-splitting hemi-laminoplasty [9] and the lamina fenestration [26]. Beside, Csaba et al. [27] have performed that different intramedullary pathologies which located in the midline of the spinal canal resection with the para-split laminotomy approach, by which certain surgical results are achieved. The common advantages of these procedures include the little influence on the original anatomical structure and biomechanical relationship of the spine, which helping the reduce of the complication occurrence such as kyphosis and cerebrospinal fluid leakage. However, as to the intramedullary tumors, larger tumors and tumors closely adhered to the spinal cord and peripheral nerve roots, these surgical methods do not provide surgeon with sufficient vision of surgical field [28]. The open-door laminoplasty not only can completely lift the lamina to help surgeon have a wide surgical field vision for the complete removal of intraspinal tumors, but also restore the anatomical structure and function of the spine after tumor resection. This approach greatly made up for the deficiency of the mentioned operation methods. Another advantage of lamina anatomical reduction is to avoid dural sac and nerve root fibrosis adhering to adjacent tissues due to the loss of normal bone coverage in the posterior wall of the spinal canal, resulting in iatrogenic spinal canal stenosis and nerve injury [3, 29].

Casha et al. [30] have reported a suspended laminoplasty technique for posterior cervical decompression and intradural access. Although the exposure range of suspended laminoplasty is wider compared with open-door laminoplasty, the segments of suspended laminoplasty need devascularization and tissue disconnection, which often leads to nonunion or delayed healing of lamina and ligaments, especially for laminoplasty involving multiple segments. Moreover, Park et al. [5] have reported that 10 patients with intra-vertebral mass undergo T-saw laminoplasty, all of which achieve satisfactory results in lamina healing. However, possible complications in the T-taw laminoplasty technique include spinal cord injury when the T-saw passes beneath the lamina, particularly in cases with larger tumors or narrower spinal canal caused by thickening of the ligamentum flavum [31]. On the contrary, open-door laminoplasty is safe to operate by cutting lamina under direct vision from outside to inside, which will greatly reduce the incidence of spinal cord injury. Besides, Casha et al. believe that the high incidence of postoperative deep infection is partly attributed to segmental devascularization after suspended laminoplasty [30]. With the open-door technique, total devascularization does not occur, which will reduce the incidence of postoperative deep infection and be beneficial to the recovery of the patients after operation [10].

There are several limitations of the current study, including small sample size and relatively short-term follow-up period. Therefore, the surgical effects of 5 and 10 years after operation need to be further verified. Overall, the surgical technique of open-door laminoplasty with ARCH plate fixation was simple, laminoplasty was less invasive on one side, and it was not limited by the patient's age, surgical site, or the number of segments.

## Conclusions

The open-door laminoplasty with ARCH plate fixation was a safe and effective surgical approach for the treatment of cervical intraspinal tumors. Besides, it had two main advantages, including a lower incidence of spinal deformities and an absence of epidural scar tissue. Moreover, the preservation of blood supply on one side of bone tissue and part of the ligament was beneficial to early healing and reduction of infection.

## Data Availability

The data used and analyzed during the current study was available from the corresponding author on reasonable request.

## Abbreviations

VAS: Visual analog scale
JOA: Japanese Orthopedic Association
MRI: Magnetic resonance imaging
CSF: Cerebrospinal fluid

## References

[1] Lee SM, Cho YE, Kwon YM (2014). Neurological outcome after surgical treatment of intramedullary spinal cord tumors. Korean J Spine.

[2] Millward CP, Bhagawati D, Chan HW, Bestwick J, Brecknell JE (2015). Retrospective observational comparative study of hemilaminectomy versus laminectomy for intraspinal tumour resection; shorter stays, lower analgesic usage and less kyphotic deformity. Br J Neurosurg.

[3] Fassett DR, Clark R, Brockmeyer DL, Schmidt MH (2006). Cervical spine deformity associated with resection of spinal cord tumors. Neurosurg Focus.

[4] Zong S, Zeng G, Du L, Fang Y, Gao T, Zhao J (2014). Treatment results in the different surgery of intradural extramedullary tumor of 122 cases. PLoS ONE.

[5] Park YJ, Kim SK, Seo HY (2019). Ligament-saving laminoplasty for intraspinal tumor excision: a technical note. World Neurosurg.

[6] Song Z, Zhang Z, Ye Y, Zheng J, Wang F (2019). Efficacy analysis of two surgical treatments for thoracic and lumbar intraspinal tumours. BMC Surg.

[7] Raimondi AJ, Gutierrez FA, Di Rocco C (1976). Laminotomy and total reconstruction of the posterior spinal arch for spinal canal surgery in childhood. J Neurosurg.

[8] Oral S, Tumturk A, Kucuk A, Menku A (2018). Cervical hemilaminoplasty with miniplates in long segment intradural extramedullary ependymoma: case report and technical note. Turk Neurosurg.

[9] Lee YS, Kim YB, Park SW (2015). Spinous process-splitting hemilaminoplasty for intradural and extradural lesions. J Korean Neurosurg Soc.

[10] Iplikcioglu AC, Hatiboglu MA, Ozek E, Dinc C, Erdal M (2010). Surgical removal of spinal mass lesions with open door laminoplasty. Cen Eur Neurosurg.

[11] Wang ZC, Li SZ, Sun YL, Yin CQ, Wang YL, Wang J, Liu CJ, Cao ZL, Wang T (2020). Application of laminoplasty combined with ARCH plate in the treatment of lumbar intraspinal tumors. Orthop Surg.

[12] Bhimani AD, Denyer S, Esfahani DR, Zakrzewski J, Aguilar TM, Mehta AI (2018). Surgical complications in intradural extramedullary spinal cord tumors—an ACS-NSQIP analysis of spinal cord level and malignancy. World Neurosurg.

[13] Zadnik PL, Gokaslan ZL, Burger PC, Bettegowda C (2013). Spinal cord tumours: advances in genetics and their implications for treatment. Nat Rev Neurol.

[14] Yousefifard M, Rahimi-Movaghar V, Baikpour M, Ghelichkhani P, Hosseini M, Jafari A, Aziznejad H, Tafakhori A (2017). Early versus late spinal decompression surgery in treatment of traumatic spinal cord injuries; a systematic review and meta-analysis. Emergency (Tehran, Iran).

[15] Miyakoshi N, Kudo D, Hongo M, Kasukawa Y, Ishikawa Y, Shimada Y (2018). Intradural extramedullary tumor in the stenotic cervical spine resected through open-door laminoplasty with hydroxyapatite spacers: report of two cases. BMC Surg.

[16] Denis F (1983). The three column spine and its significance in the classification of acute thoracolumbar spinal injuries. Spine.

[17] Ogihara S, Seichi A, Iwasaki M, Kawaguchi H, Kitagawa T, Tajiri Y, Nakamura K (2003). Concurrent spinal schwannomas and meningiomas. Case illustration. J Neurosurg.

[18] McGirt MJ, Garces-Ambrossi GL, Parker SL, Sciubba DM, Bydon A, Wolinksy JP, Gokaslan ZL, Jallo G, Witham TF (2010). Short-term progressive spinal deformity following laminoplasty versus laminectomy for resection of intradural spinal tumors: analysis of 238 patients. Neurosurgery.

[19] Asthagiri AR, Mehta GU, Butman JA, Baggenstos M, Oldfield EH, Lonser RR (2011). Long-term stability after multilevel cervical laminectomy for spinal cord tumor resection in von Hippel–Lindau disease. J Neurosurg Spine.

[20] Jiang H, Russell G, Raso VJ, Moreau MJ, Hill DL, Bagnall KM (1995). The nature and distribution of the innervation of human supraspinal and interspinal ligaments. Spine.

[21] Wu CC, Jin HM, Yan YZ, Chen J, Wang K, Wang JL, Zhang ZJ, Wu AM, Wang XY (2018). Biomechanical role of the thoracolumbar ligaments of the posterior ligamentous complex: a finite element study. World Neurosurg.

[22] Samartzis D, Gillis CC, Shih P, O'Toole JE, Fessler RG (2016). Intramedullary spinal cord tumors: part II-management options and outcomes. Glob Spine J.

[23] Mohindra S, Savardekar A (2015). Unilateral hemilaminectomy: the surgical approach of choice for juxta-medullary spinal tumors. Neurol India.

[24] Parihar VS, Yadav N, Yadav YR, Ratre S, Bajaj J, Kher Y (2017). Endoscopic management of spinal intradural extramedullary tumors. J Neurol Surg Part A, Central Eur Neurosurg.

[25] Mobbs RJ, Maharaj MM, Phan K, Rao PJ (2015). Unilateral hemilaminectomy for intradural lesions. Orthop Surg.

[26] Koch-Wiewrodt D, Wagner W, Perneczky A (2007). Unilateral multilevel interlaminar fenestration instead of laminectomy or hemilaminectomy: an alternative surgical approach to intraspinal space-occupying lesions. Technical note. J Neurosurg Spine.

[27] Padanyi C, Vajda J, Banczerowski P (2014). Para-split laminotomy: a rescue technique for split laminotomy approach in exploring intramedullary midline located pathologies. J Neurol Surg Part A Central Eur Neurosurg.

[28] Naganawa T, Miyamoto K, Hosoe H, Suzuki N, Shimizu K (2011). Hemilaminectomy for removal of extramedullary or extradural spinal cord tumors: medium to long-term clinical outcomes. Yonsei Med J.

[29] Cemil B, Tun K, Kaptanoglu E, Kaymaz F, Cevirgen B, Comert A, Tekdemir I (2009). Use of pimecrolimus to prevent epidural fibrosis in a postlaminectomy rat model. J Neurosurg Spine.

[30] Casha S, Engelbrecht HA, DuPlessis SJ, Hurlbert RJ (2004). Suspended laminoplasty for wide posterior cervical decompression and intradural access: results, advantages, and complications. J Neurosurg Spine.

[31] Kawahara N, Tomita K, Shinya Y, Matsumoto T, Baba H, Fujita T, Murakami H, Kobayashi T (1999). Recapping T-saw laminoplasty for spinal cord tumors. Spine.

